# Electroacupuncture promotes nerve regeneration and functional recovery in rats with spinal cord contusion through the coordinate interaction of CD4 and BDNF

**DOI:** 10.1002/ibra.12055

**Published:** 2022-07-26

**Authors:** Bao‐Lei Zhang, Xi‐Liang Guo

**Affiliations:** ^1^ Department of Anatomy Jinzhou Medical University Jinzhou Liaoning China; ^2^ Department of Experimental Zoology Kunming Medical University Kunming Yunnan China

**Keywords:** brain‐derived neurotrophic factor (BDNF), CD4, electroacupuncture, nerve regeneration, spinal cord injury (SCI)

## Abstract

To explore the effect of electroacupuncture on spinal cord injury (SCI) involving immune‐related factors and regeneration‐related factors in rats. The model of spinal cord contusion was established by PCI 3000 instrument. Two types of acupuncture points were selected for electroacupuncture treatment on rats. The rats were tested once a week, and the fiber remodeling was detected by magnetic resonance imaging. Transcriptome sequencing was performed on spinal scar samples. Using Python to write code, statistical analysis and bioinformatics analysis of the correlation between transcriptome sequencing data and fiber reconstruction results are carried out. Lastly, the expression of CD4 and brain‐derived neurotrophic factor (BDNF) in spinal cord scar was verified by quantitative reverse‐transcription polymerase chain reaction (qRT‐PCR). Electroacupuncture exhibited a positive effect on the recovery of motor function in rats after SCI. Bioinformatics analysis found a direct interaction between CD4 and BDNF. Transcriptome sequencing and PCR results verified that electroacupuncture significantly reduced the expression of CD4, and increased significantly the expression of BDNF, simultaneously corresponding to nerve regeneration in rats with SCI. Our results showed that electroacupuncture intervention in SCI rats improves neural behavior via inhibiting the expression of CD4 and increasing the expression of BDNF.

## Graphical abstract

Flow chart of the study. At present, the nerve regeneration repair and treatment for spinal cord injury (SCI) patients is still in an exploratory stage, and EA has received much attention as a representative treatment for traditional Chinese medicine (TCM), but its molecular mechanism of action on functional recovery and nerve repair of SCI has not been fully elucidated. We established an SD rat spinal cord contusion (SCC) model, treated with electroacupuncture for 7 weeks, and performed Basso‐Beattie‐Bresnahan (BBB) score every Sunday. Rats were subsequently subjected to MRI, fiber reconstruction, and transcriptome sequencing of spinal scar samples. Using Python to write code, statistical analysis and bioinformatics analysis of the correlation between transcriptome sequencing data and fiber reconstruction results are carried out. Lastly, the expression of CD4 and brain‐derived neurotrophic factor (BDNF) in spinal cord scar was verified by quantitative reverse‐transcription polymerase chain reaction (qRT‐PCR). RatsThe correlation analysis of transcriptome sequencing results with the number of reconstructed spinal cord fiber bundles revealed that the changes in expression of inflammatory factors represented by CD4 showed a strong negative correlation with the number of fibers, and the bioinformatics prediction revealed that there was a direct interaction between CD4 and a series of molecules of neural regeneration represented by brain‐derived neurotrophic factor (BDNF). Therefore, it is inferred that the electroacupuncture treatment in spinal cord‐injured rats may promote the regeneration of spinal cord fibers and recovery of motor function by reducing the inhibition of CD4 expression and thus enhancing the expression of BDNF.

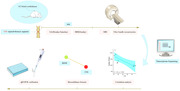

## 1 INTRODUCTION

Spinal cord injury (SCI) refers to any trauma in the central nervous system of the spine, which is a kind of nervous system disease with permanent loss of sensation, movement, and other functions in the acute and chronic stages of injury, and it is a life‐threatening condition. SCI usually has a great impact on patients' lives and psychology. Data from the National SCI Statistics Center in 2019 showed that the number of patients with SCI was approximately 291,000, and of these, 78% were male, and over time, the average age of patients with SCI is also changing from young to old, which places a great economic burden on society.^1^ Spinal cord contusion (SCC) is a common type of injury, which often occurs in accidents, such as falling from high altitudes and traffic accidents. The clinical treatment of SCI mostly depends on the severity of symptoms and the recovery of neurological function. The treatment mainly focuses on protecting patients' complete neurological function and inhibiting complications. Because of the poor nerve regeneration ability and central nervous system plasticity, nerve regeneration and repair in patients with SCI is still in an exploratory stage, and there is no effective treatment for SCI recovery.

With the emergence of more and more cases of intractable difficult and complicated diseases cured by traditional Chinese medicine (TCM), the whole world has begun to pay attention to and study the theoretical system of TCM treatment.^2^ Acupuncture as a treasure‐class treatment in China has been used to treat neurological diseases, such as stroke, Parkinson's disease, epilepsy, and trigeminal neuralgia. On the basis of acupuncture at acupoints of patients, electro‐acupuncture is used to stimulate the position of the needle handle with micro‐waves of human biological electricity, and to treat diseases by double stimulation of acupuncture and electric current at acupoints. At present, studies have shown that electroacupuncture can promote the recovery of motor and sensory functions in patients with SCI.^3^, ^4^ The study by Liu et al.^5^ indicates that electroacupuncture treatment can promote functional recovery in SCI rats and reduce tissue loss and neuronal apoptosis after SCI. In the aspect of protecting neuron survival and promoting axonal regeneration, Xu et al.^4^ reported that NT‐3 increased after electroacupuncture stimulation promoting neuronal survival, axonal regeneration, and synaptic maintenance in spinal cord neurons after SCI. Furthermore, the study by Huang et al.^6^ showed that electroacupuncture stimulation can mediate the protective effect of electroacupuncture on nerve myelin sheath by promoting oligodendrocyte increment and oligodendrocyte death. In addition, EA could promote the oligodendrocyte of endogenous neural stem cells in SCI rats and promote the proliferation of endogenous neural stem cells.^7^ Meanwhile, electroacupuncture at the two acupoints of Dazhui and Mingmen in rats with SCI could regulate the expression of NR1 and NR2, which plays a critical role in cell death following early SCI, thus contributing to the protective effect against damage.^8^ To sum up, EA can promote functional recovery and nerve regeneration after SCI by influencing the environment of the injured site of the spinal cord, reducing cell death, promoting cell proliferation and axon regeneration, and so forth; however, the molecular mechanism of EA promoting functional recovery and nerve fiber regeneration in SCI rats has not been fully clarified.

This study observed that the motor function recovery and nerve regeneration of rats with SCI treated by electroacupuncture explored its potential network mechanism and extracted the core genes in functional recovery. All the findings may contribute to electroacupuncture in the clinic and understanding the underlying molecular network mechanism.

## 2 METHODS

### 2.1 Experimental animals and grouping

Thirty healthy adult female Sprague–Dawley rats weighing 250–300 g were purchased from the Kunming Medical University Animal Center. The rats were kept in a cage at an ambient temperature of 23 ± 2°C and a natural light‐dark cycle during which they were given adequate food and water. All rats were randomly divided into three groups (*N* = 10 in each group): Sham Operation Group (Sham Group), Spinal Cord Injury Group (SCC group), and Spinal Cord Injury + Electroacupuncture group (SCC + EA Group). All experiment procedures were approved by the Animal Care & Welfare Committee of Kunming Medical University (kmmu2019005).

### 2.2 Establishment of rat SCI model and postoperative nursing

The establishment of a rat SCI model was carried out by means of an automatic hitting device in rats.^9^, ^10^ In detail, 30 Sprague–Dawley rats were weighed and anesthetized with 10 ml/kg 3.6% chloral hydrate intraperitoneally. When the rats were in a deep coma, the hair and skin were removed from the upper and lower thoracic vertebrae on the 10th. Rats were placed in a prone position on a superclean experimental operating table and were sterilized with Iodophor at the site of preparation for incision, with the T10 thoracic spinous process as the incision center, a 1–1.5 cm skin incision was made along the median of the back of the rats. After the incision, the upper fascia and some muscles were passively separated and cut off layer by layer. With the spine as the center, the two muscles were cut with a scalpel, and the spinous process and lamina of T9‐11 are fully exposed, and the spinous process and part of the lamina of T9‐11 are chewed out. Carefully, the surrounding blood vessels and nerves are avoided and the dura is exposed to remain intact as the area of injury. The rats were then transferred to a PCI‐3000 percussion device with a 2 mm diameter round head and a speed of 4 m/s, a depth of 1.5 mm, and a residence time of 0.1 s; first, the position of the percussion head at the midline of the dura surface (depth 0) was adjusted, and the computer‐controlled percussion device struck the dura mater and spinal cord tissue of the rats. The successful criterion was the obvious hematoma in the spinal cord and the sudden rigidity reaction in the hind limbs. After bleeding was stopped immediately, a suture of the muscle layer and skin layer was performed. In the Sham group, the model was established by cutting off the T9‐11 spinous process and part of the vertebral lamina and exposing the spinal cord.

To perform Postoperative Care,^9^, ^10^, ^11^, ^12^ the rats were placed on a thermostatic heating blanket to keep body temperature and wait for awakening. The respiratory condition was observed at all times before the rats were fully awoken, and the disappearance of the movement of both hind limbs was considered the criterion of successful modeling. Then the rats were transferred to a rat cage to eat and drink water normally and were given a certain amount of sunflower seeds every week to supplement nutrition. During the first 3 days, all rats were injected with 80,000 units of penicillin sodium intraperitoneally from the second day after the operation to control infection. Meanwhile, the rats were given bladder massage once a day in the morning and once a day in the evening to assist in micturition until the rats were able to urinate spontaneously. Generally, the bedding was changed every 3 days to keep the cage dry and clean. During the experiment, if the rats had wet lower body and edema and ulceration of lower limbs, they were washed with warm water, dried and wiped with iodophor, and if they tore each other's back wounds or lower limbs, they were firstly treated with H_2_O_2_ and then kept in separate cages.

### 2.3 Electroacupuncture treatment

The rats were fixed on the fixed plate in a prone position, and the limbs and tails were fixed with adhesive tape. The needles were inserted into the fixed points.^13^, ^14^, ^15^ On the first day, the upper point was selected at Dazhui (GV14) and Jiaji points on both sides of the first thoracic vertebra, and the lower point was selected at mingmen (GV4) and Jiaji points on both sides of mingmen (GB 14) On the second day, the upper part of the acupuncture points was selected to the Yang (GV9) and its bilateral Jiaji points, and the lower part was selected to the XUANSHU (GV5) and its bilateral Jiaji points. The acupoints were used in cycles according to the number of days, and the depth of needling was about 4–5 mm. Electroacupunture instrument selected sparse wave, frequency of 1, the upper side of the needle connected to the positive pole, the lower side of the needle connected to negative pole, the left side of the needle connected to the positive pole, the right side of the needle connected to the negative pole, the stimulation intensity was adjusted with reference to the rat does not hiss and the back muscles or needle handle slightly twitching as the limit, continuous EA for 10 min, and then reverse electrode stimulation for 10 min.

The corresponding acupoints were Dazhui (GV14), MingMen (GV4), Zhiyang (GV9), and XuanShu (GV5). Large vertebrae were located between the 7th cervical vertebra and the 1st thoracic vertebra; mingmen, was located between the 2nd lumbar vertebra and the 3rd lumbar vertebra; Zhiyang was located between the 7th thoracic vertebra and the 8th thoracic vertebra; hanging axis was located between the L1 and L2 vertebrae.

### 2.4 Basso‐Beattie‐Bresnahan (BBB) score

The BBB score^16^ was recorded in a closed environment at room temperature of 20° in which each rat requiring the score was left to crawl freely, and in a scoring group consisting of three experienced raters, the scoring process was double‐blind. The rats were carefully observed and the movement of the rats' hind limbs was recorded according to the BBB rating scale. Referred to the BBB score system, the hind limb movement of rats was divided into 22 grades in which the hind limb that rats could not move autonomously was 0, and the hind limb movement function of rats that was completely normal was 21, according to the function between the two were defined as 1–20 points. The scoring was performed weekly until the end of the 7th week during the survival period following SCI.

### 2.5 Magnetic resonance imaging (MRI)

The experiment was conducted at the nuclear magnetic department of Sichuan University. All MRI scanning operations were performed on a 7.0 t MRI scanner (Bruker Biospec 70/30). First, the site of SCI was found and marked with a marker pen. Then, the rats were put into the anesthesia box, and the anesthesia machine was adjusted to 3.5°. The rats were anesthetized about 1 min later. Then the anesthetized rats were placed in the supine position of the MRI machine with the mark on the back facing the + position of the machine, the mouth of the rats was fixed at the interface of the anesthesia machine, and the rats were fixed with adhesive tape, the patch of the heart rate monitor was placed on the lower abdomen of the rats and fixed with moderate pressure. Finally, we pressed the f2 button, adjusted the laser location to “+,” turned off the laser, and pressed the f1 button to send rats into the machine.

According to the preliminary results, the computer operator first sets the positioning phase of the spinal cord in three directions, then the machine begins to work formally, making structural and functional images, respectively. Fiber bundle reconstruction and counting of the MRI original files were performed after the completion of the MRI inspection.

### 2.6 Samples collection

Each rat was anesthetized by intraperitoneal injection of 3.6% chloral hydrate at a dose of 10 ml/kg. When the anesthesia depth was reached, the rats were placed in a supine position, the abdominal wall was opened, and so were the diaphragm and chest cavity, then, the heart was exposed, perfusion needle was inserted into the aorta from the left ventricle, and the needle was fixed. The right atrial appendage was open to connect to the prepared infusion set and fill with precooled saline until the liver turns white and the irrigation fluid from the right atrial appendage clears. To harvest the spinal cord, the pulp cavity was then opened, and the scar sites of SCI were removed, rinsed with normal saline, and placed into 1.5 ml PE tubes respectively, then moved into a −80°C refrigerator for temporary preservation.

### 2.7 Eukaryotic messenger RNA (mRNA) sequencing

The concentration and purity of total RNA were detected by nanodrop 2000, the integrity of RNA was detected by agarose gel electrophoresis, and the value of Rin was determined by Agilent2100 Using magnetic beads with Oligo (DT) and Ploya to carry out A‐T base pairing, the mRNA was separated from the total RNA, and the enriched mRNA was added into fragmentation buffer. The fragment of mRNA was reverse‐transcribed to form a single‐stranded complementary DNA (cDNA) by reverse transcription with random primers, followed by two‐stranded synthesis to form a stable double‐stranded structure, and a base was then added to the 3′‐end for joining the y‐shaped adapter; the product after the adapter was purified and fragment sorted, and polymerase chain reaction (PCR) amplification of the sorted product was performed to purify the final library; subsequently, on‐line sequencing by Illumina HiSeq was performed.

### 2.8 Analysis of sequencing information

Data quality control statistics were performed on raw data from Illumina sequencing results, and raw sequencing data were filtered to obtain high‐quality sequencing data; in RNA‐Seq analysis, rSEM was used to calculate gene expression levels by the number of clean reads (reads counts) mapped to genomic regions. The FPKM values (Fragments Per Kilobase per Million mapped reads) are used as indicators of gene expression levels. After obtaining the number of Read Counts of genes, differential expression analysis was performed for Sham vs. SCC and SCC vs. SCC+EA.

### 2.9 Correlation analysis between gene expression and MRI data

The expression data of all genes in spinal cord scar tissue obtained by genomics were matched with the fiber number data of the corresponding rats, and then the association between gene expression and the number of spinal fibers was found by correlation analysis between the two groups of data. Because of the huge amount of data analysis here, data analysis is accomplished by Python programming. In Python, pandas library is used for one‐to‐one data extraction, pairing, splicing, and data export. The RE library is used to match the data names in the process of data extraction and data splicing. The scipy database was then used to judge the normal distribution of the data and analyze the correlation statistics (Pearson correlation analysis was used for the normal distribution of the data, and Spearman correlation analysis was used for the nonnormal distribution of the data). Finally, the results of statistical analysis are derived in the form of tables. Set the condition of *p* < 0.01 to filter the data, and sort the *r*‐value column, that is, the correlation coefficient column, according to its absolute value from large to small.

### 2.10 Screening of coexisting differentially expressed genes

A personalized screen was performed for differential gene expression in the Sham VS SCC Group and SCC VS SCC + EA Group in the sequencing results, setting screening conditions FDR < 0.01, |log2fc|>1.5; After the abovementioned screening conditions are completed, the FDR values are sorted from small to large, and all the gene data that meet the screening conditions in the file are obtained. The final gene list was put into Venny 2.1 (https://bioinfogp.cnb.csic.es/tools/Venny/) for intersection selection, that is, genes with specific expression in two different groups were selected, and the results of intersection gene data were saved. Download the colorful style of the Venny picture.

### 2.11 Protein ‐ protein interaction (PPI) network construction, function and pathway enrichment analysis

To further explore the functions of differentially expressed genes and their interactions, the String database (https://String-db.org/) was used to construct the PPI network of differentially expressed proteins and download the PPI network interaction table data results. Using Cytoscape 3.7.2 software, after importing the interaction table, we set up different size nodes and different thickness connecting lines according to Degree value and combined value to draw the PPI network diagram of Hub gene, by using the third‐party tool cytohubba in the software to screen out the top 10 degree targets.

The Metascape database (http://Metascape.org/) was used for differential expression protein GO functional enrichment and KEGG pathway enrichment analysis with a *p*‐value cutoff of 0.05. The first 20 terms were selected according to the Enrichment Score (Es) from the largest to the smallest, and the top 20 terms were selected according to the *p*‐value from the smallest to the largest, and visualized separately by applying the Bioinformatics platform (http://www.bioinformatics.com.cn). 

### 2.12 Construction of differential gene volcano map and Hub gene expression heatmap

After the result table file of gene expression difference analysis is imported into the R language, the corresponding working path was set, and the table file was called with openxlsx package. After setting the scatter color, plate size, and up‐down division conditions, we use the GGPLOT2 package to draw the differential gene volcano map. The average expression levels of different genes were summarized in a table with the same gene pairs. After calling the table file using the openxlsx package, the expression data in the table were normalized using a log‐transformed approach, and then the gene expression heat map was drawn using the corresponding color gamut set by the PHEATMAP package in R language.

### 2.13 Searching and screening for genes related to nerve regeneration

Using the GeneCards database (https://www.genecards.org/), we typed the keyword “Nerve regeneration” into the search box, search for genes associated with nerve regeneration, then clicked Export and select Excel to download the results from the website into the table. In the Genecards database, the higher the Relevance score value, the stronger the association between the factor and nerve regeneration, the genes were selected as the result of the screening of nerve regeneration‐related genes from the top to the bottom after the descending order of the Relevance score column, and a total of 200 differentially expressed genes were combined with the final screened set.

### 2.14 Quantitative reverse‐transcription PCR (QRT‐PCR)

Total RNA of 0.5 cm spinal cord scar segments was extracted with TRIzol reagent (Takara Bio Inc.) and reverse‐transcribed into cDNA using the Revert Aid kit (Thermo) according to the reference protocol provided by the reagent manufacturer. Primers for immune‐related factor CD4 and growth factor BDNF were designed using Primer 5.0 software and designed by Takara Biotechnology Co., Ltd. The sequence is shown in Table 1. The reaction system is shown in Table 2. This cDNA was then used as a template and glyceraldehyde 3‐phosphate dehydrogenase (GAPDH) as an internal reference to amplify the cDNA using the Revert Aid kit. In the first cycle, step 1 was predenatured by 95°C for 5 min, Step 2 was denatured by 95°C for 10 s, and Step 2 was denatured by 95°C for 10 s The third step, 54°C, was annealed for 20 s, and the fourth step, 72°C, was annealed for 20 s. The threshold period (CT value) of each sample was recorded, and the second cycle to the 40th cycle was started from the second step, the relative expression amount was calculated using the $2^{-∆∆C_{t}}$ method, and the GAPDH value was normalized.

**Table 1:** List of nucleotide sequences of genes and primers used in PCR experiments

Genes	Forward	Reverse
GAPDH	CAAGGCTGAGAATGGGAAGC	GAAGACGCCAGTAGACTCCA
CD4	AAGGCTCCTTCTTCCCAGTC	GCCAGAACCAGCAAACTGAA
BDNF	ATCGAAGAGCTGCTGGATGA	GTTTGCGGCATCCAGGTAAT

Abbreviations: BDNF, brain‐derived neurotrophic factor; GAPDH, glyceraldehyde 3‐phosphate dehydrogenase; PCR, polymerase chain reaction.

**Table 2:** Reaction systems used in PCR experiments

Name of reagent	Amount used (μl)
Beatar SybrGreen qPCR mastermix	10
PCR Forward Primer (10 μM)	0.5
PCR Reverse Primer (10 μM)	0.5
DNA templates	1
ddH_2_O (sterilized distilled water)	8.0
Total	20

Abbreviation: PCR, polymerase chain reaction.

### 2.15 Statistical analysis

SPSS 25 software package, Python 3.8 software, and related third‐party libraries (re, pandas, Scipy) were used to analyze the data. The difference analysis data were expressed as $\bar{x}$ ± s, and the correlation analysis data were expressed as *p* and correlation coefficient *R*. The statistical methods used in the difference analysis included repeated measures analysis of variance and one‐way analysis of variance. The comparison between LSD and Dunnett was considered statistically significant (*p* < 0.05) The statistical methods used in correlation analysis included Pearson correlation analysis and Spearman correlation analysis.

## 3 RESULTS

### 3.1 Therapeutic effect of electroacupuncture for SCI in rats

**Figure 1:**
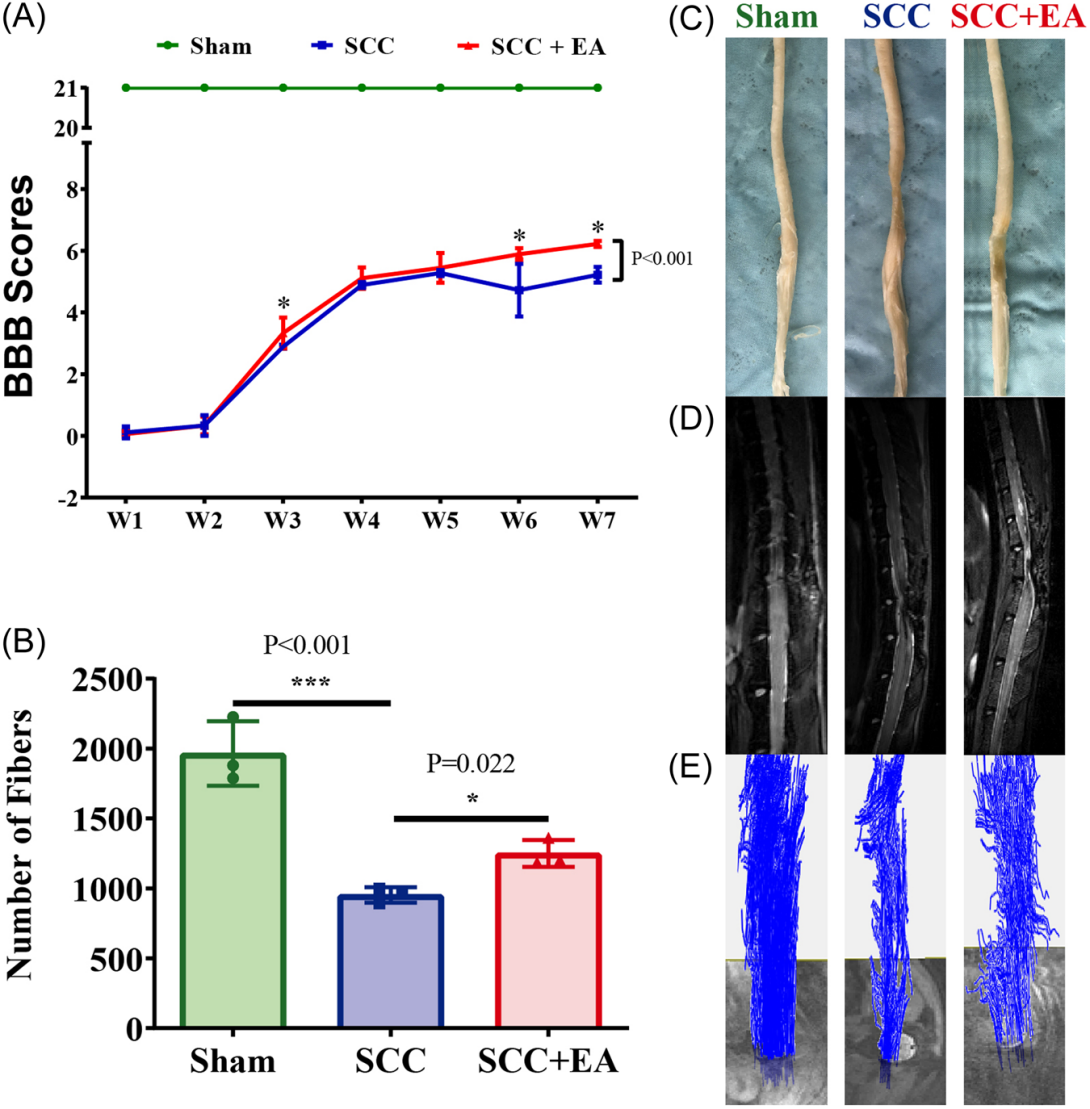
Evaluation of therapeutic effect of electroacupuncture on spinal cord injury in rats. (A) After EA, the BBB score of each group was changed (*p < 0.05). There was a significant difference between SCC + EA Group and SCC Group. Statistical test: repeated measures analysis of variance. The error bars extend to the most extreme data points, in the 1.5‐fold interquartile range. (B) After 7 weeks of EA, the number of MRI fibers in the spinal cord of the rats in each group was significantly different (*p < 0.05, ***p < 0.001). There were significant differences between the Sham group and SCC Group, SCC + EA Group, and SCC Group. Statistical test: one‐way ANOVA. The error bars extend to the most extreme data points, in the 1.5‐fold interquartile range. (C) Spinal cord tissue samples of rats in each group. (D) The anatomical image of the spinal cord in each group. (E) Microbeam reconstruction of the spinal cord in rats of each group. ANOVA, analysis of variance; BBB, Basso‐Beattie‐Bresnahan; MRI, magnetic resonance imaging; SCC, spinal cord contusion. [Color figure can be viewed at wileyonlinelibrary.com]

Studies have shown that electroacupuncture at “Dazhui” and “Mingmen” and “Jiaji” points has an effective therapeutic effect on SCI in rats.^17^, ^18^ BBB is a common behavioral method that can directly reflect the recovery degree of SCI by scoring the limbs of rats. It has important reference significance for this experiment. Therefore, the BBB score of each group of rats was carried out weekly after rehabilitation after a series of interventions (Figure 1A). The BBB score of the Sham group was always the highest score at 21 points, the BBB score was affected by time after SCI. The BBB score in SCC and SCC + EA groups increased gradually with the increase of multiple BBB scores, which indicated that the BBB score in the SCC Group and SCC + EA Group increased gradually with time, and the motor ability of hind limbs in both groups recovered to different degrees. After SCI, the BBB score of the SCC Group was significantly lower than that of the Sham group; compared with the SCC Group, the BBB score of the SCC + EA Group was significantly higher than that of the SCC group, especially at the 3rd, 6th, and 7th week. It is suggested that EA can promote the recovery of the motor ability of hind limbs in SCI rats. However, its treatment course may be a chronic one, so the difference in BBB scores in the first 5 weeks is statistically significant only in Week 3. The results showed that there was an interaction between EA treatment and time. With the increase in scores, the increase of BBB score was different in the SCC + EA Group, and the increase of BBB score was higher in the SCC + EA Group than in the SCC Group.

At the same time, after the 7th week, we scanned the SCI rats with MRI, then analyzed the results and reconstructed the spinal cord with the original fMRI data by using the software, which can directly observe and compare the fiber repair and regeneration of spinal cord directly. Tissue loss at the site of SCI (middle segment) was severe in the SCC group compared with the Sham group (Figure 1C,D), and the number of spinal cord fibers was significantly reduced (*p* < 0.001) (Figure 1B,E); it is suggested that the injury of the spinal cord leads to the loss of normal spinal cord tissue and the breakage of spinal cord fibers and the decrease of the number of spinal cord fibers. Tissue defects at the site of SCI (middle segment) were restored in the SCC + EA group compared with the SCC group (Figure 1C,D), with a significant increase in the number of spinal cord fibers (*p* = 0.022) (Figure 1B,E); it is suggested that EA stimulation after SCI may promote the regeneration of injured spinal cord tissue and the repair of spinal cord fiber tract.

### 3.2 Differentially expressed genes after EA treatment and the correlation with the amount of spinal cord fiber reconstruction in SCI rats

**Figure 2:**
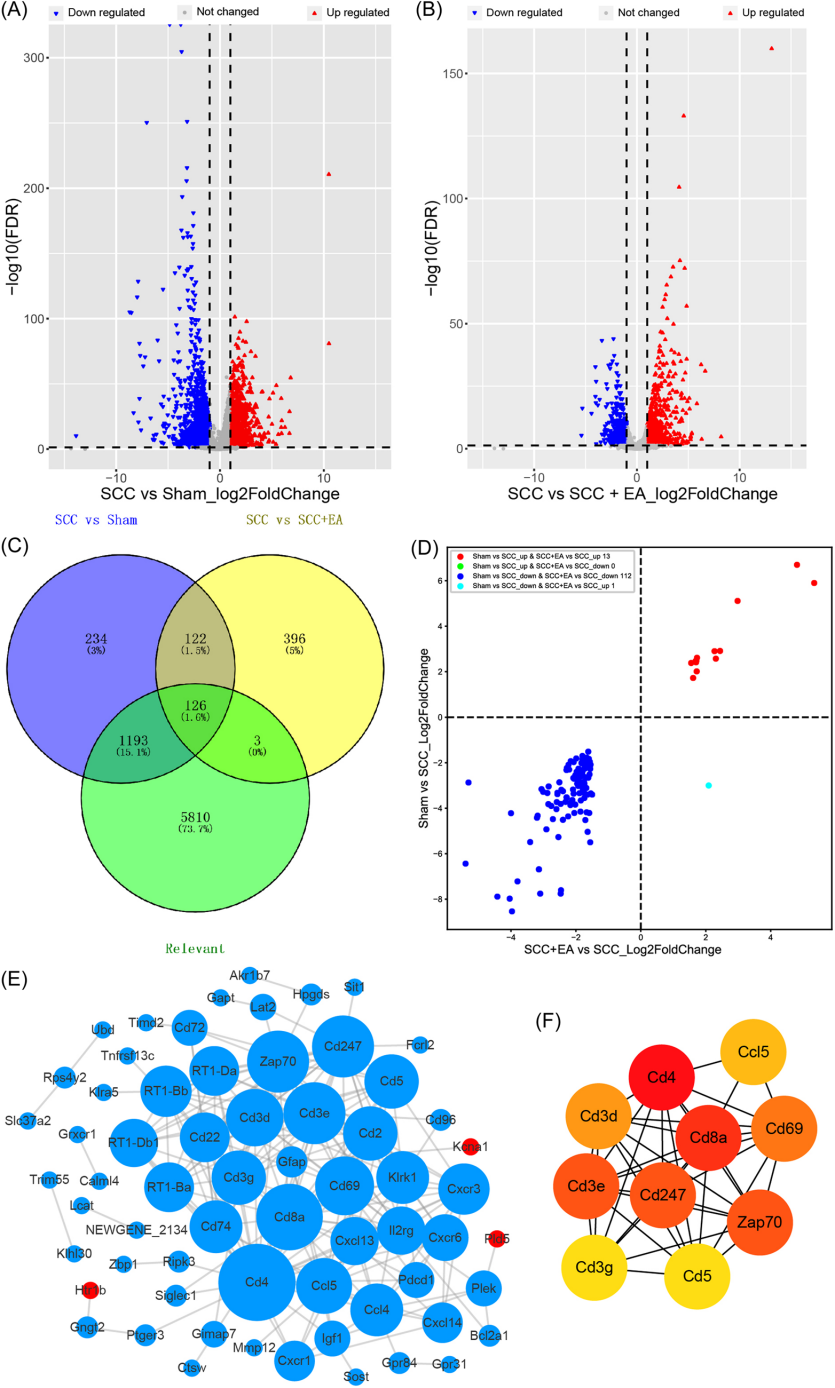
Differentially expressed genes in the transcriptome of spinal cord injury rats in each group. (A) and (B) Volcano plots of gene expression changes (A: gene expression changes between Sham and SCC groups; B: gene expression changes between SCC and SCC + EA Groups; blue inverted triangle represents downregulated genes, the red positive triangle represents upregulated genes, and gray dots represent unchanged genes). (C) Venny map of differentially expressed genes between SCC Group and SCC Group, SCC Group and SCC + EA Group, transcription group, and the number of spinal cord fibers reconstructed. (D) Four‐quadrant plot of the correlation between mRNA expression and the number of reconstructed spinal cord fibers in SCC Group and SCC Group, SCC Group, and SCC + EA Group. The red dots are all upregulated proteins, dark blue dots show both downregulated proteins, green dots show Sham/SCC differentially upregulated and SCC + EA/SCC differentially downregulated proteins, and light blue dots show Sham/SCC differentially downregulated and SCC + EA/SCC differentially upregulated proteins. (E) Protein interplay plots of Venny intersection data of differentially expressed genes between SCC group and SCC group, SCC group, and SCC + EA group, transcriptome and reconstructed spinal fiber count correlation analysis results. The red nodes were all upregulated proteins, dark blue nodes were all downregulated proteins, green nodes were SCC/Sham differentially upregulated and SCC + EA/SCC differentially downregulated proteins, light blue nodes were SCC/Sham differentially downregulated and SCC + EA/SCC differentially upregulated proteins, the degree gradient shows that the larger the node, the greater the degree value, and vice versa. (F) Protein target interaction graph of the top 10 degrees, the higher the degree value is, the redder the color. mRNA, messenger RNA; SCC, spinal cord contusion. [Color figure can be viewed at wileyonlinelibrary.com]

To investigate the effect of electroacupuncture on gene expression in SCI of rats, we extracted the spinal cord tissue and sequenced the transcriptome. By collating and analyzing the sequencing data of the scar tissue of the rat spinal cord, we set the screening criteria for significantly differentially expressed genes as follows: FDR < 0.05 and |log2fc|≥1. A total of 3788 differentially expressed genes were screened in the Sham group compared with the SCC Group; 1180 were upregulated and 2608 were downregulated (Figure 2A). A total of 1443 differential genes were screened in the SCC Group compared with the SCC + EA Group, of which 858 were upregulated and 585 were downregulated (Figure 2B). We then proceeded to set the precise screening criteria as FDR < 0.01 & |log2fc|≥1.5, with a total of 1679 differential genes screened in the Sham group, compared with the SCC Group, and a total of 647 differential genes screened in the SCC group compared with the SCC + EA Group.

After the correlation analysis between the sequencing data of the rat spinal cord and the fiber reconstruction data of the functional magnetic resonance imaging (fMRI) of the rat spinal cord, a total of 7150 differentially expressed genes were found, of which 1908 were positively related genes, there were 5242 negatively correlated genes. These are related differentially expressed genes and crossed with the previously screened 1679 Sham versus SCC groups and 647 SCC versus SCC + EA groups, the cross‐sectional genes, that is, the differential genes present in both the Sham and SCC groups and the differential genes between the SCC group and the SCC + EA Group, were selected, and the spinal cord fMRI fiber reconstruction data of the SCI rats were compared with the relevant genes in a total of 126 genes (Figure 2C). Among them, there were 13 SCC upregulated genes in Sham versus SCC, 112 SCC + EA versus SCC downregulated genes in Sham versus SCC, and 1 SCC + EA vs SCC upregulated gene in Sham versus SCC (Figure 2D). The protein interaction analysis of these 126 genes and the screening of hub genes (Figure 2E) showed that 64 proteins with interaction contained three upregulated genes and 61 downregulated genes, there were 208 PPI relationships, and the top 10 interacting protein genes were CD4, Cd8a, Cd3e, ZAP70, CD247, CD69, Cd3d, CCL5, CD5, and CD3G, respectively, ranked by degree (Figure 2F). It is suggested that EA intervention may promote the recovery of motor function and the regeneration of spinal cord fibers by stimulating the related factors of immune function.

### 3.3 Protein interaction analysis and correlation analysis between neural regeneration and related gene set in SCI rats

**Figure 3:**
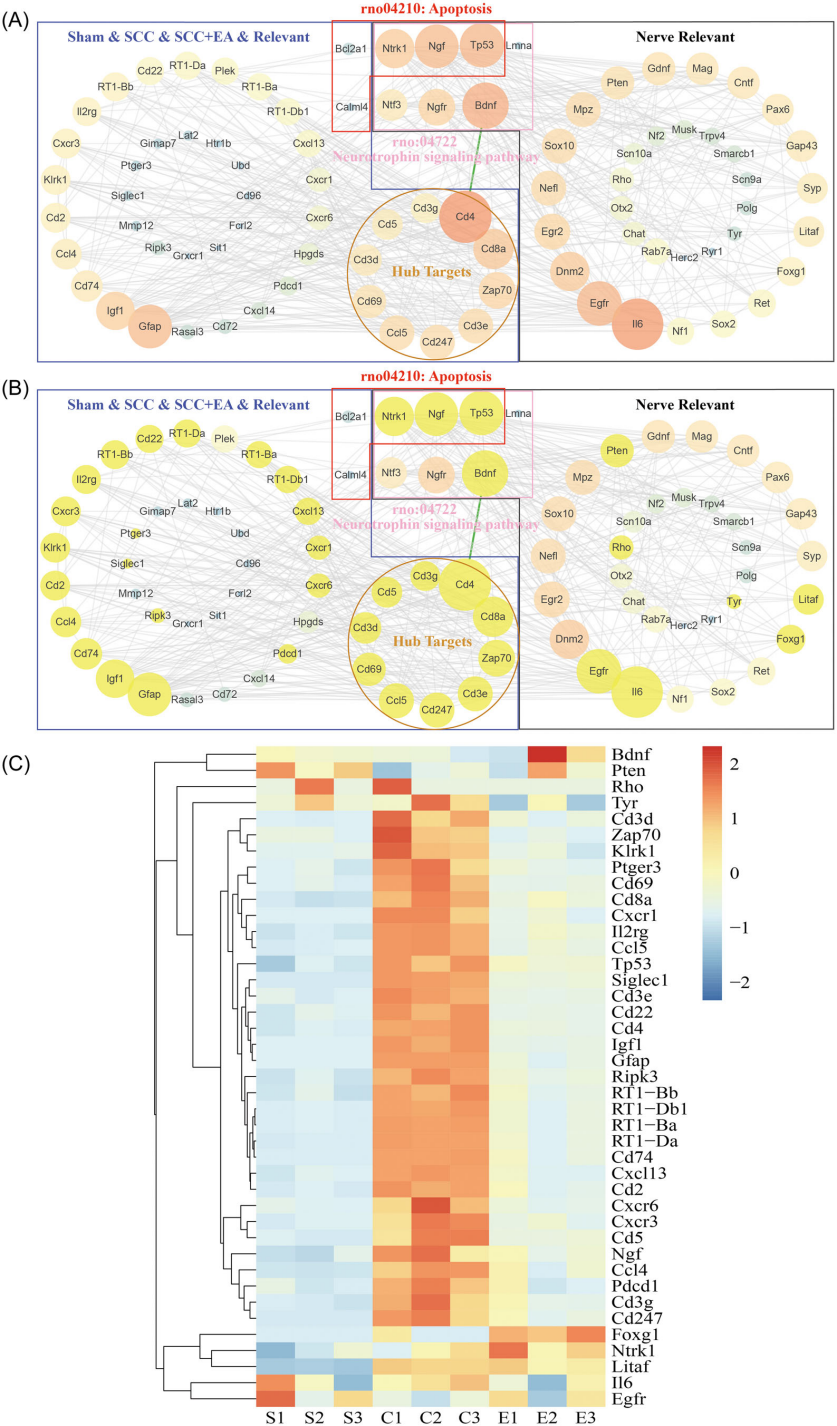
Protein interaction analysis of neural regeneration gene set with data from spinal cord injury rats. (A, B) Protein interaction network diagram. The blue box is the result of the intersection of three groups of data and correlation analysis, the black box is the related factor of nerve regeneration, and the red box is the factor enriched into the apoptotic signal pathway The pink box is the factor that is enriched into the Neurotrophin pathway, the orange circle is the Hub factor, and the yellow node in figure B is the factor that interacts directly with CD4. The green line is the line where CD4 interacts with BDNF. (C) Heatmap of expression changes of all interaction factors with CD4. BDNF, brain‐derived neurotrophic factor. [Color figure can be viewed at wileyonlinelibrary.com]

The first 74 groups of the above 126 differentially expressed genes and nerve regeneration‐related genes were selected, and the final list of 200 targets was obtained. The interaction map was optimized by Cytoscape software, and 141 protein interactions were found, with 692 PPI relationships between them. The Cytoscape software was used to screen out 10 protein targets in Figure 2F. At the same time, we screened 10 protein targets that were directly linked to each other (Figure 3A), the neural regeneration‐related targets with direct interaction with the most core target CD4 were BDNF, TP53, NGF, NTRK1, IL6, EGFR, PTEN, Litaf, Foxg1, Rho, and Tyr (Figure 3B), and a heatmap of expression changes was plotted (Figure 3C). The expression levels of BDNF, EGFR, and TP53 in the SCC group were significantly lower than those in the SCC + EA Group. The expression levels of CD4, Cd8a, Zap70, Cd3e, CD247, CCL5, CD69, Cd3d, CD5, and Cd3g in SCC group were significantly higher than those in SCC + EA group. BCL2A1 and CALML4 in Sham & SCC & SCC + EA & Relevant are enriched in the apoptosis signaling pathway with TP53, NTRK1, and NGF, which are related to nerve regeneration, meanwhile, TP53, NTRK1, NGF, NTF3, NGFR, LMNA, BDNF, which are related to nerve regeneration, were enriched in Neurotrophin signaling pathway. These results suggest that EA intervention may be performed by stimulating related factors of immune function and through apoptosis signaling pathway in SCI rats, to affect the expression of nerve growth‐related factors, such as BDNF and Neurotrophin signaling pathway, and then promote the regeneration of spinal cord fibers in rats.

### 3.4 Combinatorial analysis and the function and pathway enrichment analysis of SCI‐related gene sets in rats

To enrich the function of the final protein, the 141 proteins were analyzed for KEGG pathway enrichment and GO Biological process (BP), Cellular component (CC), and Molecular function (MF) enrichment using the Metascape database.

**Figure 4:**
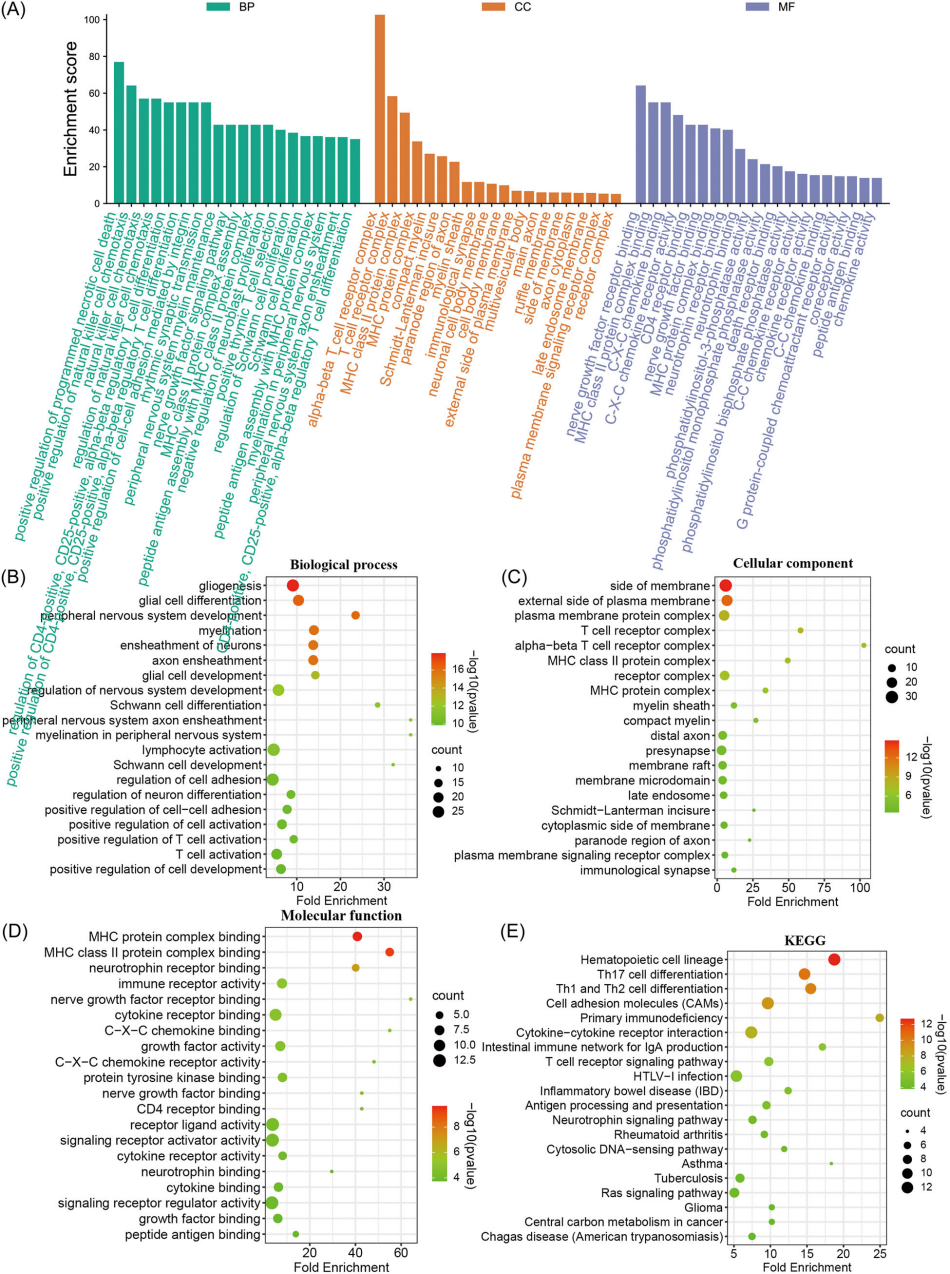
Protein GO function enrichment and KEGG pathway enrichment in spinal cord injury rats. (A) A three‐in‐one histogram of the top 20 terms in the GO‐BP, GO‐CC, and GO‐MF categories, sorted by ES values from large to small: GO‐BP is shown in green, GO‐CC is shown in orange, and GO‐MF is shown in purple. (B–E) The bubble diagram shows the first 20 terms enriched by GO‐BP, GO‐CC, GO‐MF and the first 20 terms enriched by the KEGG pathway, selected from small to large by *p*‐value, the dot size shown in the figure represents the number of proteins enriched by this term. BP, Biological process; CC, Cellular component; GO, Gene ontology; KEGG, Kyoto Encyclopedia of Genes and Genomes; MF, Molecular function. [Color figure can be viewed at wileyonlinelibrary.com]

The first 20 BP, CC, and MF terms for GO, filtered by ES from large to small, are shown in Figure 4A. The terms with the highest concentration of GO‐BP, GO‐CC, and GO‐MF were positive regulation of programmed necrotic cell death, alpha‐beta T‐cell receptor complex, and nerve growth factor receptor binding, respectively.

Most of the proteins in the GO‐BP results screened according to *p*‐value from small to large were mainly enriched in gliogenesis, glial cell differentiation, peripheral nervous system development, myelination, ensheathment of neurons, and other functions (Figure 4B). Most of the proteins in GO‐CC were concentrated on the side of the membrane, external side of the plasma membrane, plasma membrane protein complex, T cell receptor complex, and alpha‐beta T‐cell receptor complex (Figure 4C). Most of the GO‐MF proteins were concentrated in MHC protein complex binding, MHC class II protein complex binding, neurotrophin receptor binding, immune receptor activity, nerve growth factor receptor binding, and other nerve growth factor receptor functions (Figure 4D). This may mean that EA intervention can promote the development of the immune and nervous systems in SCI rats. The results of the KEGG pathway analysis showed that most of the proteins were concentrated in the signaling pathways Hematopoietic cell lineage, Th17 cell differentiation, Th1 and Th2 cell differentiation, Cell adhesion molecule adhesion molecules (CAMS), and Primary Primary immunodeficiency, and so forth (Figure 4E), the results of GO analysis were similar to those of GO analysis, suggesting that EA intervention may promote the recovery of motor function and the regeneration of spinal cord fibers by regulating cell differentiation and immune function.

### 3.5 The expression of CD4 and BDNF in Sham Group, SCC Group, and SCC + EA Group were verified after 7 weeks

**Figure 5:**
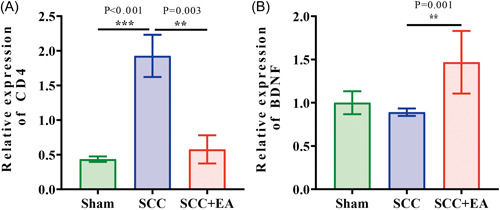
mRNA expression levels of CD4 and BDNF in different subgroups. The green column is the Sham group, the blue column is the SCC Group, and the red column is SCC + EA Group. (A) The expression of CD4 in Sham, SCC, and SCC + EA groups and(B) the expression of BDNF in Sham, SCC, and SCC + EA groups. The results are presented as mean ± standard deviation. Data were statistically assessed using one‐way analysis of variance, with error bars extending to the most extreme data points, that is, 1.5‐fold interquartile range, **p < 0.01; ***p < 0.001. mRNA, messenger RNA; SCC, spinal cord contusion. [Color figure can be viewed at wileyonlinelibrary.com]

As mentioned in the above results, the final analysis of the sequencing results of the different subgroups of spinal cord scar tissue resulted in the interaction between Cd4‐represented immune‐related factors and BDNF‐represented neural regeneration‐related factors, the results of sequencing analysis indicated that Cd4 expression increased sharply after SCI and decreased significantly after electroacupuncture treatment, and its Degree value was the highest among the protein interactions in the correlation analysis (Figure 3E,F), meanwhile, Bdnf expression, which had direct interactions with Cd4 (Figure 4B,C), decreased rapidly after SCI and increased significantly after electroacupuncture treatment. Based on this, we selected two molecules, Cd4 and Bdnf, for validation using qRT‐PCR; the results showed that the expression of CD4 was significantly increased in the SCC group compared with the Sham group (*p* < 0.001), and in the SCC + EA group compared with the SCC group, the expression of CD4 in SCC + EA group decreased significantly to a level close to that of Sham group (*p* = 0.003) (Figure 5A), suggesting that EA treatment may repair the immune disturbance in SCI rats. The expression of BDNF in the SCC + EA group was significantly higher than that in the SCC group (*p* = 0.001) (Figure 5B), suggesting that EA treatment may promote the increase of expression of nerve regeneration‐related factors in SCI rats.

## 4 DISCUSSION

Spinal cord injuries can cause serious complications, including pain sensitivity, permanent loss of sensation, motor function, and so on, ranging from paraplegia to life‐threatening injuries, bringing a heavy burden to patients and society.^19^ It has been reported that electroacupuncture can promote the recovery of sensory and motor functions after SCI.^14^, ^20^ However, the mechanism of electroacupuncture has not been clearly explained. Wei et al.^13^ showed that EA could improve motor function in SCI rats and concluded that EA could improve motor function in rats by inhibiting PTEN and P53 expression. In the present study, we used the BBB score to evaluate the motor function in SCI rats, and the results showed that EA did improve the motor function of the hind limbs of SCI rats, which was consistent with previous studies. At the same time, MRI results showed that EA reduced the loss of spinal cord tissue and promoted the regeneration of nerve fibers in SCI rats, min‐Fei Wu et al. observed the changes in protein levels in the early stage and late stage of acute SCI in EA and found that EA could decrease the expression of RhoA and Nogo‐A mRNA and protein; it also reduces neuronal apoptosis at the site of SCI and promotes regeneration of spinal cord fibers, repair of spinal cord tissue, and revive neural function.^21^ A study by Li et al.^22^ confirmed that electroacupuncture treatment of SCI rats had higher orientation and parallelism of fluorescein‐positive nerve fibers than the injury group, indicating that electroacupuncture can promote nerve fiber regeneration in rats. This is the same as our results, but the mechanism of EA promoting nerve fiber regeneration is not clear.

Electroacupuncture transcutaneous nerve stimulation is now recognized by the World Health Organization (WHO) and National Institutesutes Health (NIH) in the United States, and it has been used clinically to suppress pain and control inflammatory responses in various human diseases.^23^ Recent studies have shown that electroacupuncture intervention can suppress peripheral inflammation‐induced Fos expression in the spinal cord, control pain and allergy, and reduce inflammatory responses, Lao et al. electro‐acupuncture is parameter‐dependent and point‐specific in its anti‐hyperalgesia, and selection of suitable parameters and points can selectively inhibit FOS expression in the superficial layer of the spinal dorsal horn and activate Fos expression in a deeper layer^24^ Dai et al. found that EA at “Changqiang” acupoint in rats with SCI significantly reduced spinal cord hemorrhage and reduced the inflammatory response to the SCI, thereby reducing the attack in neuronal cells.^25^ In his research on chemotherapy‐induced peripheral neuropathy, Lu pointed out that EA can alter axonal degeneration by inhibiting inflammatory signaling pathways and attenuating the overexpression of the TRPV1 receptor, thus inhibiting inflammatory responses and reducing neuropathic pain.^26^ Fang et al. found that the motor function of rats was restored and autophagy was upregulated after EA pretreatment and EA postinjury in spinal cord ischemia‐reperfusion injury rats; this reduced apoptosis and neuroinflammation.^27^ We analyzed the transcriptome of the scar tissue of the SCI rats and finally concluded that after EA, the final Hub genes were CD4, CD8A, ZAP70, Cd3e, CD274, CCL5, CD69, Cd3d, CD5, and CD3G. The expression levels of these immune‐related factors were significantly increased in the injury group but significantly decreased in the EA Group. At the same time, GO and KEGG results indicated that all factors in the final PPI network were significantly enriched in the immune process‐related functions and pathways, suggesting that EA may act on these immune‐related factors, through immune‐related functions and pathways, and can control the abnormal immune response, control the inflammatory reaction, and promote the repair of SCI. Zhang et al.^28^ showed that EA could significantly reduce the expression level of CD4 in the perioperative model rats and accelerate the recovery to the control level, suggesting that EA could improve the immune dysfunction of the rats and promote the recovery of immune function in rats.^28^ Wang et al., by establishing a model of delayed‐type hypersensitivity disease, found that electroacupuncture at “Znli” (ST 36) reduced the percentage of CD4 + IFN‐γ + T cells, thereby exerting its anti‐inflammatory effect.^29^ In our sequencing and PCR results, the expression of CD4 was significantly increased in the SCC group compared with the Sham group and significantly decreased in SCC + EA group compared with the SCC group to resolve the Sham level; therefore, we conjectured that EA treatment on SCI rats may control the excessive immune response by decreasing the expression of CD4 in the rats, to bring it back to the normal level, thereby promoting the recovery of SCI.

BDNF expression and its functional implication BDNF is a Neurotrophin that stimulates the growth of neurites, protects neuronal survival and maintenance,^30^ promotes neuronal development and regeneration, and promotes the proliferation of neural stem cells, improving the growth capacity of axons^31^ is an important signaling molecule that activates the downstream signaling cascade of NTRK2.^32^ Studies have found that BDNF protects axonal‐transected neurons that are not mature and, more specifically, BDNF prevents massive death of motor neurons following transected sciatic nerve axons in neonatal rats.^33^ Exogenous BDNF can promote the regeneration of DRG neurons and sensory axons after sciatic nerve injury, and enhance the regeneration of ascending sensory neurons.^34^ In addition to reducing neuronal death, BDNF also promotes neuronal axonal regeneration. A study by Kobayashi et al. noted that BDNF treatment increased the number of axonal rsns regenerated by peripheral nerve grafts implanted at the transverse site of the neck several‐fold. It was concluded that the application of appropriate nutritional factors could attenuate these effects of the axonal transaction, thereby enhancing the ability of damaged cells to maintain axonal regeneration.^35^ By transplanting BDNF‐expressing fibroblasts into adult rats with unilateral complete hemi‐transverse injury to the third cervical medullary segment (C3), Ying Jin et al. observed the regeneration of axons in the red spinal tract (RST), reticulospinal tract (REST) and vestibular spinal tract (VST) after 4 weeks of injury using BDA tracing, along with the recovery of motor function in the forelimbs of rats.^36^ Analysis of biological processes in our GO functional analysis found that BDNF is enriched in areas involved in the neurodevelopmental function. The results of sequencing showed that the expression level of BDNF in the SCC + EA group was significantly higher than that in the SCC group, which was also confirmed by PCR results; EA can promote the recovery of the injured spinal cord and increase BDNF expression, which provides a suitable microenvironment for nerve development and spinal cord fiber regeneration.

The interaction between CD4 as a representative of immune‐related factors and BDNF as a representative of neuroregeneration‐related factors has been reported in the literature to exert anticancer effects. The results of the study by Xiao et al. reported that a rich environmental profile is an important factor in cancer risk and progression, the enhanced anticancer effect of the enriched environment is mediated in part by the regulation of immune responses, such as CD4 helper T cells and CD8 cytotoxic T lymphocyte; BDNF acts as a key brain mediator and synergizes with immunity to exert anticancer effects.^37^ In the study of sepsis‐associated encephalopathy, Luo found that the precursor form of BDNF is proBDNF, which is upregulated in the immune system by downregulating circulating CD4 + T cells, limiting their infiltration into the meninges and disrupting meningeal pro/anti‐inflammatory homeostasis, promotes the pathogenesis of sepsis‐associated encephalopathy.^38^ The neurological disorder of the interaction between CD4 and BDNF has also been reported. Gold et al.^39^ found that testosterone treatment significantly reduced the percentage of CD4 + T cells in patients with testosterone‐induced multiple sclerosis; it also increases the production of BDNF and exerts its potential neuroprotective effects.^39^ Kerschensteiner et al. showed through their study that activated human CD4 + T cell lines secrete bioactive BDNF in vitro, thereby supporting the survival of neurons in vitro. It is therefore postulated that the production of BDNF by activated human T cells plays a neuroprotective role in inflammatory encephalopathies.^40^ In a study of SCI, Wang et al. noted that CD4 + T cells in spinal cord tissue 8 days after SCI secrete BDNF and NT‐3, and found the ability of A91‐DC to induce T‐cell proliferation. At the same time, it enhances the ability of T cells to secrete BDNF and NT‐3, thus providing a stable microenvironment during the repair of SCI.^41^ Similarly, our results showed that after 7 weeks of EA treatment, the expression levels of CD4, an important immune‐related factor, were significantly decreased compared with those in the SCC group; compared with the SCC Group, the expression of BDNF was significantly increased, and CD4 was found to be the key factor in the treatment of SCI by electroacupuncture, and it had direct interaction with BDNF, which is also the core factor in the related factors of nerve regeneration. It is preliminarily indicated that electroacupuncture treatment of SCI in rats by affecting the interaction between CD4 and BDNF, inhibition of inflammatory reaction, and promotion of nerve regeneration play a role in the treatment of SCI. The deep regulation mechanism of CD4 and BDNF in SCI needs further study. In our study, the first prediction of the bioinformatics results was the CD4 represented by the 10 immune‐related factors, after adding nerve regeneration‐related factors to predict the interaction of protein, it was found that there was a direct interaction between CD4 and BDNF. These results indicated that EA intervention may promote the regeneration of spinal cord fibers by reducing the expression of CD4 and increasing the expression of BDNF.

To sum up, in this study, we observed that electroacupuncture improved motor function and promoted the increase of spinal nerve fibers in SCI rats; at the same time, bioinformatics prediction results and PCR results indicated that this may be the effect of EA stimulation on the coexpression of CD4 and BDNF in SCI rats, and the interaction between them, thus inhibiting the inflammatory reaction and promoting nerve regeneration, so it played a role in SCI repair.

## AUTHOR CONTRIBUTIONS

Xi‐Liang Guo contributed to the conceptual framework and revised the manuscript. Bao‐Lei Zhang is responsible for the experimental design, operation, data collection, data curation, data statistics, graphic production, writing, and revision.

## CONFLICT OF INTEREST

The author declares no conflict of interest.

## TRANSPARENCY STATEMENT

All the authors affirm that this manuscript is an honest, accurate, and transparent account of the study being reported; that no important aspects of the study have been omitted; and that any discrepancies from the study as planned (and, if relevant, registered) have been explained.

## ETHICS STATEMENT

All experiment procedures were approved by the Animal Care & Welfare Committee of Kunming Medical University (kmmu2019005).

## Data Availability

The materials used during the current study are available from the corresponding author on reasonable request.
